# A Multi-Objective Approach for Drug Repurposing in Preeclampsia

**DOI:** 10.3390/molecules26040777

**Published:** 2021-02-03

**Authors:** Eduardo Tejera, Yunierkis Pérez-Castillo, Andrea Chamorro, Alejandro Cabrera-Andrade, Maria Eugenia Sanchez

**Affiliations:** 1 Grupo de Bio-Quimioinformática, Universidad de Las Américas, Quito 170513, Ecuador; raul.cabrera@udla.edu.ec; 2 Facultad de Ingeniería y Ciencias Aplicadas, Universidad de Las Américas, Quito 170513, Ecuador; andrea.chamorro@udla.edu.ec (A.C.); mariaeugenia.sanchez@udla.edu.ec (M.E.S.); 3 Escuela de Ciencias Físicas y Matemáticas, Universidad de Las Américas, Quito 170513, Ecuador; 4 Carrera de Enfermería, Facultad de Ciencias de la Salud, Universidad de Las Américas, Quito 170513, Ecuador

**Keywords:** preeclampsia, multi-objective models, drugs repurposing, machine learning

## Abstract

Preeclampsia is a hypertensive disorder that occurs during pregnancy. It is a complex disease with unknown pathogenesis and the leading cause of fetal and maternal mortality during pregnancy. Using all drugs currently under clinical trial for preeclampsia, we extracted all their possible targets from the DrugBank and ChEMBL databases and labeled them as “targets”. The proteins labeled as “off-targets” were extracted in the same way but while taking all antihypertensive drugs which are inhibitors of ACE and/or angiotensin receptor antagonist as query molecules. Classification models were obtained for each of the 55 total proteins (45 targets and 10 off-targets) using the TPOT pipeline optimization tool. The average accuracy of the models in predicting the external dataset for targets and off-targets was 0.830 and 0.850, respectively. The combinations of models maximizing their virtual screening performance were explored by combining the desirability function and genetic algorithms. The virtual screening performance metrics for the best model were: the Boltzmann-Enhanced Discrimination of ROC (BEDROC)_α=160.9_ = 0.258, the Enrichment Factor (EF)_1%_ = 31.55 and the Area Under the Accumulation Curve (AUAC) = 0.831. The most relevant targets for preeclampsia were: AR, VDR, SLC6A2, NOS3 and CHRM4, while ABCG2, ERBB2, CES1 and REN led to the most relevant off-targets. A virtual screening of the DrugBank database identified estradiol, estriol, vitamins E and D, lynestrenol, mifrepristone, simvastatin, ambroxol, and some antibiotics and antiparasitics as drugs with potential application in the treatment of preeclampsia.

## 1. Introduction

Preeclampsia is a complex pregnancy related disease with unknown pathogenesis. During pregnancy, preeclampsia appears after 20 weeks of gestation in two possible scenarios: early or late preeclampsia onset [1]. The worst maternal and fetal prognostic is during early manifestation of the disease, which frequently leads to pregnancy interruption or premature delivery. Currently, preeclampsia is the leading cause of fetal and maternal mortality during pregnancy. This is why not only better diagnosis strategies (early diagnosis) but also exploration of new drugs for preeclampsia management are needed.

Any drug for the treatment of preeclampsia will have, in general, two possible options (which are not necessarily independent of each other): (1) to be a preventive drug for use early in pregnancy to prevent a possible preeclampsia manifestation or (2) to be a drug capable of handling symptoms and/or disease progression. The first option includes the use of low-dose aspirin in women with high risk of preeclampsia [2], the use of magnesium sulfate [3] and the administration of vitamin D [4,5]. Drugs in the latter group could extend the gestational age, thus increasing the chances of fetal growth and maturity to facilitate a safer delivery. The first-line treatment for this approach of preeclampsia management is mainly anti-hypertensive drugs such as labetalol, hydralazine or nifedipine [1], with hypertension being a main symptom to deal with during disease progression. In any case, the effectiveness of the treatment depends on the disease progression, but eventually delivery is the only definitive treatment for preeclampsia [1,6,7].

Because we are dealing with pregnancy, de novo drug design presents several important drawbacks not only during in vitro assays but also during clinical trials. These difficulties are highlighted by the lack of clinical trials during pregnancy involving drugs discovery [8]. Therefore, exploring drugs repurposing for pregnancy associated diseases, such as preeclampsia, could be a potentially more cost-efficient and a faster solution [9].

The strategies for drug repurposing can be grouped in several ways discussed in the recent reviews by Lofti Shahreza et al., and Pushpakom et al. [10,11]. For the purpose of this study, we will name some of the most widely used strategies:

1. Target based approach. The disease target is known and this information is used for drug repurposing. These methods usually use a variety of data sources, i.e., protein-protein interaction and drug-target interaction networks.
2. Drugs-Drugs similarity based approach: The similarity between drugs is explored (through a wide variety of information like gene expression profile, possible targets, etc.) to identify new candidates.
3. Gene signature based approach (sometimes called the data driven approach [10,11]): The known gene signature generated by the disease is known. In this case, we can use the comparison between the gene signatures of the disease and drugs to identify the candidate drug.

In preeclampsia, the targets related with the disease or even its pathogenesis are unknown. More important, even if some targets are proposed considering recent research on preeclampsia, it is possible that no drugs had been previously tested in clinical trials for them. Therefore, we do not really know if that target could be desirable for a possible drug treatment. However, following this target-based approach, Shuyu Zhao et al. [12] recently identified transcription factors and genes related to methylation-mediated transcriptional dysregulation motifs in preeclampsia. Using these genes and transcription factors, they created a drug-target network and a drug repurposing was done using the centrality of the drugs in the network. On the other hand, several transcriptomic studies with microarray data are available in public databases, while some pathogenesis analyses also use this kind of data [13,14,15,16]. However, the majority of these microarrays were obtained from different regions of the placenta and not from maternal blood. From them, it could be possible to proceed with this approach. However, the implications for drug discovery of having placenta instead of maternal blood information for the definition of the gene signature are ignored. In this direction, one attempt was made to explore in silico possible drugs for preeclampsia treatment using connectivity-map (CMAP) and microarrays as the main strategy [17]. Moreover, in terms of drug repurposing for preeclampsia, only one study was found [18]. In it, the authors experimentally evaluated the effect of more than 500 drugs on the expression of the placental growth factor (PLGF) protein using the HUVE cell line as experimental model.

An alternative strategy (mixing target and ligands based approaches) can be to provide the current information for the drugs that can be safely used during pregnancy and preeclampsia. That is, (1) to extract all targets associated with the drugs currently on clinical trials for preeclampsia and (2) to combine them with the undesirable targets derived from the drugs that cannot be used during pregnancy and that are associated with the treatment of hypertension. This proposed strategy requires a multi-target and multi-objective model potentially capable of identifying drugs candidates for the treatment of preeclampsia.

## 2. Results and Discussion

### 2.1. Targets Analysis

From the 19 drugs obtained from the ClinicalTrials for preeclampsia treatment or management and from the 41 antihypertensive drugs following the ACE inhibition and angiotensin receptor antagonist mechanisms, we obtained 155 unique proteins combining DrugBank and ChEMBL information. For the 155 possible proteins, we extracted and curated (see Materials and Methods) all active/inactive compounds from the ChEMBL database. After all filtering and curation steps, we finally obtained 55 proteins and 147,456 compounds-proteins interactions. In this final dataset there are 45 target and 10 off-targets. However, from these 45 targets, 10 are common to both groups. These common proteins are: EGFR, PHG1, SLC6A2, PPARG, ADORA3, CYP2C9, CYP3A4, CYP2C19, ABCB1 and CYP2C8. This means that some drugs used in preeclampsia share targets with those used for hypertension treatment. Because all drugs are actually used in preeclampsia, these targets were included in the “target” group and removed from the “off-target” set.

All of the targets for preeclampsia are: CHRM4, PDE5A, FYN, AR, HDAC2, CACNA1C, VDR, SCN5A, IKBKB, CHRM5, ESR1, ADRB2, CHRM2, ADRB1, HTR1A, CHRM1, ADORA1, PTGS2, CASP3, OPRK1, SLC6A3, PPARA, ESR2, CHRM3, ADORA2A, EDNRA, CYP2D6, CYP1A2, NR1I2, NOS1, CCR2, SCN9A, NOS2, CASP1 and NOS3. On the other hand, the final list of off-targets is: ACE, ERBB2, BCHE, F2, CES1, AGTR1, REN, MMP9, MMP2 and ABCG2.

**Figure 1:**
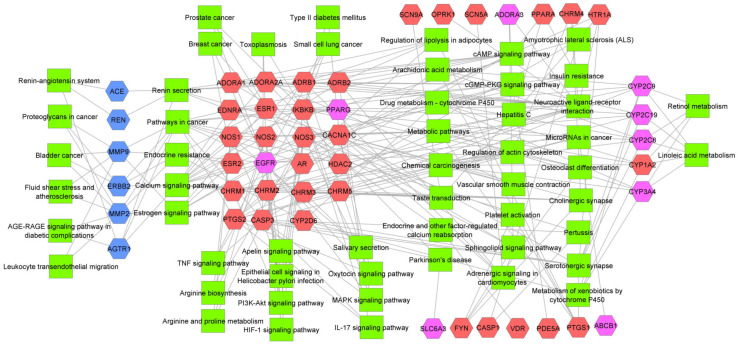
Network between proteins (target—red, off-targets—blue, common targets—purple) and enriched metabolic pathways (green).

In order to comprehend the relationship between the 55 proteins with preeclampsia and hypertension, we performed a pathway enrichment analysis. The analysis showed that “Pathway in Cancer” and “Renin Secretion”, “Endocrine resistance”, “Calcium signaling pathway” and “Estrogen signaling pathway” are common between targets and off-target genes while the Renin-Angiotensin system is mainly enriched with off-targets (Figure 1). A total of 23 proteins were associated with pathways common to targets and off-target proteins. In this group, only 2 (EGFR and PPARG) were explicitly identified as common proteins between targets and off-targets proteins during our initial analyses. We could notice several enriched pathways related to cancer in several forms (i.e., prostate cancer, pathway in cancer, small cell lung cancer, chemical carcinogenesis, etc.). The enrichment of pathways in cancer is not surprising. Preeclampsia had been previously molecularly associated with cancer mainly because their common relationship with angiogenesis [13,19,20]. Diseases like toxoplasmosis and diabetes, which are mainly enriched in the target group, are also well-known to be related to preeclampsia [12,21,22,23,24].

Several metabolic pathways that were detected as being enriched by our 55 proteins had also been found in previous bioinformatics analyses of transcriptomic data in preeclampsia. The HIF-1 signaling pathways are known to be involved in preeclampsia. Actually, placenta hypoxia (probably associated with endothelial dysfunction) is one of the events involved in the proposed mechanism of preeclampsia pathogenesis [13,14]. Other detected pathways that had been previously found through microarray analysis of preeclampsia vs normal placenta are the TNF signaling pathways [13,14], pathways in cancer [13], the MAPK signaling pathway [12,13,14] and even diabetes and vascular smooth muscle contraction [14]. All of these pathways are mainly connected to target proteins instead off-targets. These findings suggest that even when we are going through a very reduced protein space (compared to transcriptomic analysis), some of the metabolic pathways associated with preeclampsia are conserved.

Moreover, the enrichment analysis clearly indicates that hypertension related pathways are also in contact with proteins related to the preeclampsia group (i.e., calcium signaling, renin secretion and linoleic acid metabolism). This means that some drugs could have an effect (which is probably unknown) on different proteins and metabolic pathways. This polypharmacological mechanism implies that some drugs used for hypertension treatment though the ACE inhibition could also affect, for example, pathways in diabetes, TNF signaling or even arginine metabolism. More importantly, from the space of the desirable proteins in preeclampsia (targets) some of them could have different risk levels to interlink with undesirable targets (off-targets). So, the complexity of the disease as well as the necessity of avoiding undesirable pathways interconnected through desirable targets, support our strategy of using a multi-objective modeling approach. In other words, we need to find desirable drugs while reducing their risk to interact with undesirable targets.

### 2.2. Models and Early Recognition Ability

**Figure 2:**
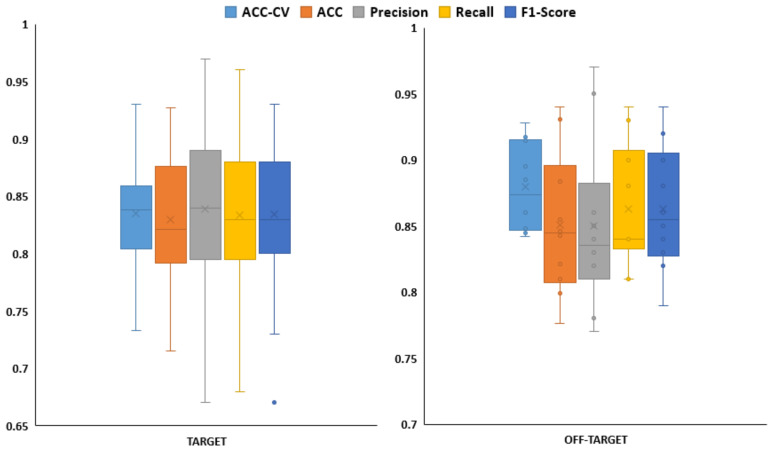
Distribution of performance metrics obtained for all models for targets (**Left**) and off-targets (**Right**). ACC-CV: accuracy values obtained in cross validation analysis. All other metrics (accuracy, precision, recall and f1-score) were obtained for the external dataset.

The goal of this research is to find molecules that selectively inhibit targets from the preeclampsia group. Using the TPOT pipeline modeling strategy, we found predictive models for each of the 55 proteins. The final list of the obtained models with all the performance metrics is available in Supplementary Materials (Table S1). Figure 2 presents the distributions of the performance metrics for all the models obtained for all targets and off-targets.

The average accuracy for the off-target models in cross-validation analysis was found to be ACC-CV = 0.880 while ACC = 0.851 in the external data of the same group. Moreover, the average values for precision and recall in the external data were 0.850 and 0.863, respectively. Regarding the models for the targets in preeclampsia similar results to the off-targets were found with ACC-CV = 0.835 and ACC = 0.829 when predicting the external dataset. The average values for precision and recall in the external data for this group of targets were 0.838 and 0.833, respectively. Detailed information for each model can be found in Supplementary Materials (*tpot_CHEMBLID.zip*).

Once the 55 models were obtained, we needed to go through multi-objective model construction. This needed to prioritize desirable targets and avoid undesirable ones. However, we did not know a priori the influence of each protein toward a preeclampsia drug that avoids interactions with off-targets. In order to determine this, we ran the genetic algorithm for feature selection (models/proteins selection) in order to obtain the optimal set of proteins for the virtual screening. The optimization problem needed to combine the different targets (using the desirability function) in a way so that the virtual screening prioritizes the drugs from the clinical trials related to preeclampsia (19 drugs in 14,686 total compounds). The genetic algorithm population comprises 4000 models that evolved for 3000 generations. The fitness function used in the genetic algorithms was the maximization of BEDROC values. The enrichment parameters of the best performing model were: Boltzmann-Enhanced Discrimination of ROC (BEDROC) = 0.258, the Enrichment Factor (EF)_1%_ = 31.55 and the Area Under the Accumulation Curve (AUAC) = 0.831. This result means that the best virtual screening model is capable of enriching the top 1% of the ranked list with desirable drugs almost 32-fold more than a random model.

**Figure 3:**
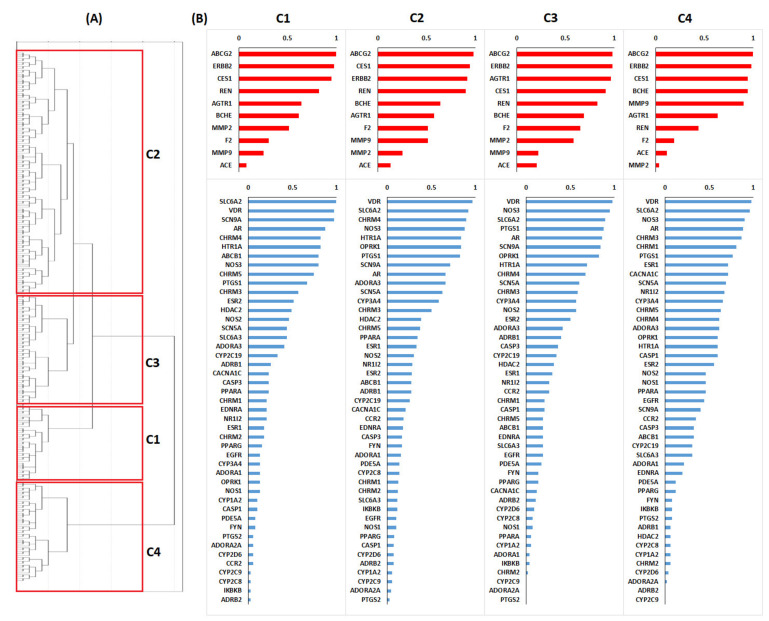
(**A**) Dendrogram obtained from all virtual screening performance models with BEDROC > 0.15. (**B**) Clusters are labeled as **C1** to **C4**. The relevance (S_i_) of the Targets (blue) and Off-Targets (red) was computed for each cluster.

Even when the best model is good, different models (especially those closer to it in BEDROC values) could identify different combinations of targets and off-targets for accomplishing a desirable screening performance. Therefore, instead of using the best model, we selected the top 275 models (all models with BEDROC ≥ 0.15) for the evaluation of the targets’ relevance. We expected in this multi-objective model that desirable targets as well as undesirable targets would be differently selected and consequently that their relevance could change. Because different models could provide different sets of selected targets, and because an individual analysis of each of the 275 models is computationally expensive, we first performed a cluster analysis to group similar models (Figure 3A). Basically, models selecting the same group of targets and off-targets will be more similar to each other and would be grouped in the same cluster.

Considering the obtained dendrogram, we selected four clusters of models. All models in each cluster were used to score the frequency of selection of the preeclampsia targets and off-targets. In this context, the amount of models selecting preeclampsia targets or off-targets was considered to be a measure of their significance for the early recognition of desirable drugs (Figure 3B).

Each cluster of models did not provide exactly the same ranking of targets/off-targets. However, they were very similar. We performed another grouping of models using 7 clusters instead of 4 (data not shown) that provided similar results to those obtained with 4 clusters. Among preeclampsia targets, AR and VDR were always selected among the top 5 targets in all clusters. Progesterone and estrogen receptors are known to be involved in preeclampsia pathogenesis [25] and vitamin D is considered as an effective preventive treatment [5,26]. Similarly, ESR1, ESR2, CHRM4 and CHRM3 were also regularly highly ranked in the four clusters. ESR1 and ESR2 are related to sexual hormone metabolism. On the other hand, SLC6A2 (together with MMP11 and IL18BP) had been recently found to be an early predictor of preeclampsia [27]. These results indicated that hormones and vitamin D related drugs would probably be enriched in the molecular virtual screening. Additionally, it is quite relevant that some of these highly relevant targets share some metabolic pathways with the off-target group (see the enriched metabolic pathway in Figure 1). Models in cluster 1 prioritize SCN9A which is a sodium channel, and its role is unknown in preeclampsia. On the other hand, models in clusters 2 and 4 prioritize NOS3. This target is related with nitric oxide metabolism, which is known to play an important role in preeclampsia [28,29]. Interestingly, Dong et al. recently found that 2-methoxyestradiol stimulates NOS3 activity [30], thereby providing a link between hormone-related and nitric oxide metabolisms.

In the group of off-targets, it is interesting to note that REN is better ranked than ACE. Actually in clusters 1 to 3, REN is among the top prioritized off-targets. Similarly, ABCG2 (the most relevant in the four clusters) is involved in bile secretion. Polymorphisms in ABCG2 had been associated with uricemia [31], which in turn is associated with preeclampsia [32]. Moreover, recent works on ABCG2 had related it with trophoblast invasion [33] and even with the risk of preeclampsia in a Chinese population [34]. The epidermal growth factor receptor 2 (ERBB2) is in some way similar to EGFR (which is ranked at a low level in preeclampsia) and was highly ranked in the off-targets group, as well as CES1 which is mainly involved in detoxification of xenobiotics and some lipids’ metabolism. These proteins have not been very well studied under conditions involving preeclampsia. Matrix metalloproteins, specially MMP9, were also selected in model 4 and in some cases were more relevant than MMP2. Previous studies indicate that MMP9 is increased in hypertensive patients [35] and decreases with a antihypertensive treatment [36]. However, in pregnancy, the increment in the levels of MMP2 and MMP9 is related to placentation and uterine expansion [37,38]. Specially, in the case of MMP9, this increment is probably related to the estrogen and progesterone production during pregnancy [38]. Therefore, it is interesting that our models (especially those in cluster 2) were chosen to avoid interactions with this type of receptors.

In the process of optimization through genetic algorithms to finally obtain the multi-objective model (or models in our case), each of the 55 models predicted the probability of the 14,686 molecules being active/inactive against all 55 proteins (see *ScreeningData.txt* Supplementary Materials). These probabilities were then combined by the models obtained with the genetic algorithm. In general, the majority of those compounds did not have experimental evidence of interaction with more than 1 or 2 proteins (similarly to the drugs used for repurposing). Moreover, following the previous discussion regarding possible polypharmacological actions, we could note that in general, our multi-objective models prioritize drugs capable of interacting with AR, VDR, SLC6A2, ESR1, ESR2 and/or NOS3. At the same time, these drugs also need to have a reduced probability to interact with REN, ABCG2, ERBB2 and/or MMP9. This polypharmacological mechanism of action is the core function of multi-objective modeling.

Because of the complexity of preeclampsia, a set of druggable targets to be used for the treatment of the disease was unknown. Therefore, we considered traditional target-based modeling approaches (molecular docking, molecular dynamics, etc.) to be unpractical at this point. Repurposing strategies based on gene signatures had been successfully applied for repurposing topiramate [11] and statins [39]. However, this repurposing strategy based on gene signature of drugs and diseases is not suitable for preeclampsia because gene expression studies in preeclampsia mainly focus on the placenta.

As previously discussed, several proteins in the target space derived from the drugs related to preeclampsia management (Figure 1 and Figure 3) enrich metabolic pathways common to those previously obtained in gene enrichment analyses for preeclampsia. This indicates that, despite the uncertain match between the gene signature in preeclampsia and the gene signature of drugs, we can use our 55 models to define a compound-protein interaction network or target interaction signature of all drugs used in preeclampsia management. A similar idea based on a multi-target approach obtained from system biology was successfully applied to identify statins as potential therapies in chronic lymphocytic leukemia in vitro [40]. In preeclampsia, the recent work of Shuyu Zhao et al. [12] also identify a set of targets which later involve a drug-target network approach for repurposing. The work of Shuyu Zhao et al. [12], which is mainly focused on the study of transcription factors and related genes in preeclampsia, uses a list of drugs that extends beyond only including the approved ones. In those works, the identification of the potential targets was done through a bioinformatics and/or system biology approach instead of a set of known drugs, as in our study. Additionally, for the creation of our target interaction signature, we included undesirable targets because they also have a possible teratogenic effect. Because these proteins are naturally connected with desirable interactions, they could lead to undesirable drugs. We consider this last point to be a step forward toward the inclusion of the undesirable effects of drugs in repurposing efforts against preeclampsia.

### 2.3. Drug Repurposing

**Figure 4:**
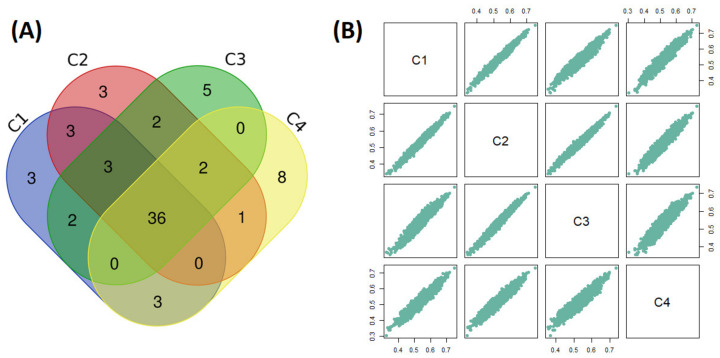
(**A**) Venn diagram of the top 50 drugs obtained for consensus models in each cluster. (**B**) Correlogram of the global desirability values of all drugs across the four clusters. The average correlation coefficient is 0.962 (0.935–0.982).

Considering that each cluster (even when not being very different) could probably prioritize different molecules, we decided to perform the repurposing across each cluster. Thus, the global desirability (GD_i_) of each repurposed drug was obtained in each cluster. Therefore, in order to explore the general similarities of the desirability values between each cluster, a correlogram was computed (Figure 4B).

The global desirability computed for all repurposed drugs is highly correlated between the four clusters. This indicates that the small variation in the target ranking across models do not have a strong influence on the global desirability of the drugs (Figure 4A). The top 50 molecules (about the 2% of all screened drugs) were selected from each cluster according to their global desirability values (GDi) (Figure 4A). A total of 71 unique drugs are found among the 50 best ranked across all clusters. From all these drugs, 36 were found in the four clusters (Figure 4A). The full list of ranked drugs across the four clusters is available in Supplementary Materials (Table S2).

The 36 drugs common to all clusters are: ergocalciferol, flunisolide, erythromycin, cladribine, venlafaxine, amcinonide, desogestrel, phenylephrine, ivermectin, hydrocortamate, mifepristone, loperamide, nalbuphine, flurandrenolide, paricalcitol, fulvestrant, methylprednisolone, ethinylestradiol, abacavir, sibutramine, trilostane, clarithromycin, budesonide, desonide, estriol, desvenlafaxine, lumefantrine, ambroxol, ulipristal, eribulin, bromhexine, fluprednisolone, lynestrenol, pexidartinib, cyclopenthiazide and drostanolone propionate.

The other drugs that are not shared by all clusters are: calcifediol, vitamin E, cholecalciferol, azithromycin, citalopram, medrysone, imipramine, acetyldigitoxin, voriconazole, simvastatin, nystatin, hexachlorophene, roxithromycin, estradiol, disulfiram, oxymetazoline, tolterodine, selegiline, ouabain, escitalopram, calcipotriol, ergosterol, cholesterol, bithionol, plicamycin, chlorcyclizine, rolapitant, norethynodrel, lidoflazine, estradiol cypionate, estradiol valerate, DL-alpha-tocopherol, hydrocortisone valerate, paramethasone acetate and cefiderocol.

**Figure 5:**
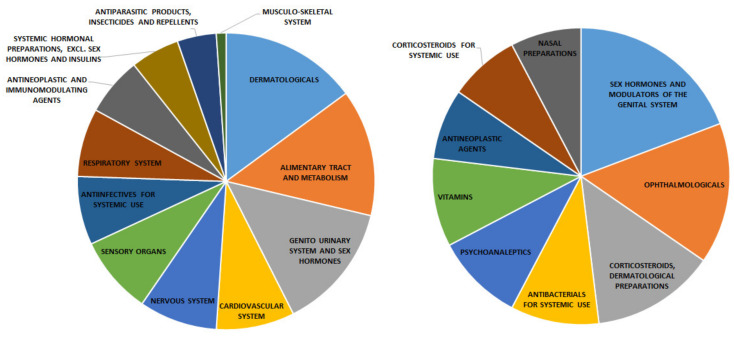
Distribution of ATC codes level 1 (**Left**) and level 2 (**Right**) from all 71 unique drugs identified by the virtual screening strategy.

The analysis of the most frequent ATC codes (Levels 1 and 2, Figure 5) for the 71 unique drugs identified through virtual screening points to the relevance of the drugs categorized as dermatological (involving usually antibacterial, antibiotics and even hormone based categories), alimentary tract and metabolism (in our data mainly composed of vitamins) and genito urinary system and sex hormone (composed mainly from hormone related categories). As dermatological and sensory organs categories (ATC level 1), ophthalmological drugs (ATC level 2) comprise a wide variety of antibacterial and corticosteroid drugs. The presence of antiparasitic, antineoplastic and inmunomodulating agents, as well as drugs involved in the respiratory system is also an interesting result. Considering the target relevance obtained by the multi-objective models (Figure 3), it seems reasonable for prioritization of the vitamins and hormone-related drugs to be achieved as shown in Figure 5.

In the group of drugs related to the respiratory system, we found four subgroups in the unique 71 drugs: (1) flunisolide and budesonide which are corticoids with anti-inflammatory effects, (2) ambroxol and bromhexine with a mucolytic effect, (3) oxymetazoline and phenylephrine which are α-adrenergic agonists and (4) chlorcyclizine which is an antihistaminic of the phenylpiperazine class (inhibitors of histamine-H1 receptor). Out of these drugs, flunisolide, budesonide, bromhexine and phenylephrine are part of the 36 drugs common to all clusters. Antihistaminic drugs do not increase the risk of fetal malformation or spontaneous abortion and could improve maternal outcomes, including a reduction of preeclampsia [38,41,42]. The involvement of histamine receptors in preeclampsia had been explored previously by other authors [43,44]. Inhaled corticosteroids can be safely used during pregnancy [41] and they are used for asthma management during pregnancy. We know that poorly controlled asthma is associated with an increased risk of preeclampsia [42,43] and that inhaled short-acting β2-agonists reduce the risk of hypertensive disorders of pregnancy in women with asthma compared to those not using them [43,44]. However, the ingestion of steroids could be possibly associated with adverse outcomes during pregnancy [45]. Despite this, no studies had been reported regarding inhaled corticosteroids. Oxymetazoline and phenylephrine are α-adrenergic agonists. Phenylephrine spinal administration causes hypotension in women with preeclampsia [46], but no studies were found about the use of these drugs for the treatment or prevention of preeclampsia with any other administration via.

The group of drugs related to cardiovascular system are: phenylephrine, cyclopenthiazide (both are found in the list of 36 common drugs), acetyldigitoxin, simvastatin, ouabain, cholesterol, lidoflazine and hydrocortisone valerate. Cyclopenthiazide is described as a type of diuretics and is also used in hypertension management. The use of diuretics during pregnancy is well known, specially nifedipine [1] which is also part of our initial group of drugs derived from clinical trials. In fact, a similar thiazide, hydroflumethiazide, is currently used for preeclampsia management [47]. On the other hand, even when the effect of simvastatin during preeclampsia in unknown, a close statin (pravastatin) is known to reduce the endothelial dysfunction associated with preeclampsia [48]. Moreover, oubain and digitoxin are capable of inhibiting sFlt-1 production [49], which is elevated in preeclampsia and is directly associated with possible preeclampsia pathogenesis mechanisms. However, no data was found of these and other drugs (found under the cardiovascular system category) regarding animal or other studies related to the prevention or management of preeclampsia.

In the group of anti-infective drugs and in our list of repurposed drugs common to all models, we found the antibiotics erythromycin, abacavir, clarithromycin, azithromycin, voriconazole roxithromycin and cefiderocol. None of these drugs, except for azithromycin, have received focus on their possible effect in preeclampsia in any previous study. Azithromycin (included in our initial 19 drugs from clinical trials) with a current clinical trial (NCT03233880) has indirect evidence of facilitating preeclampsia management [50]. Interestingly, a recent study found that doxycycline (a derivate of oxytetracycline) prevents hypertensive pregnancy in rats [51]. In fact, any maternal infection, either viral or bacterial, increases the risk of preeclampsia by around two fold [52]. Despite the use of antibiotics during pregnancy having been widely studied, their potential protective effect against preeclampsia or other pregnancy hypertensive conditions is mainly unknown. It is interesting that abacavir, an antiviral drug, appears in the list of unique drugs. In general, the administration of antiviral drugs had been associated with an increased risk of preeclampsia [53,54] but it is unclear what is the mechanism involved in this association. Regarding the antiparasitic drugs involved, we found ivermectin, lumefantrine (these two found in the common 36 drugs), disulfiram and bithionol. Ivermectin is a broad spectrum antiparasitic drug, as is bithionol to some degree. None of these drugs have provided any evidence of usefulness during preeclampsia or outcome studies during pregnancy. Interestingly, lumefantrine is used against malaria infection. The connection between malaria and preeclampsia is well known; however, no information was found regarding the preeclampsia incidence when using any of these drugs. On the other hand, disulfiram is mainly used to manage alcoholic addiction. It is an inhibitor of the alcohol dehydrogenase and is known to reduce the expression of sFlt-1, as well as being a soluble endoglin (s-Eng) through an unknown mechanism [55]. Both proteins play a major role in preeclampsia, but no further studies of disulfiram are available.

In our ATC codes (and repurposed drugs) there are other two interesting groups: genito urinary system and sex hormones (especially steroidal hormones), as well as alimentary tract and metabolism drugs (especially vitamins). The hormone imbalance during pregnancy is well known and is a reason behind the proposal of progesterone as preventive candidate (Clinical Trials NCT03297216). The identified drugs are: desogestrel, mifepristone, ethinylestradiol, estriol, ulipristal, lynestrenol (these are also found in the common 36 drugs), nystatin, estradiol, tolterodine, norethynodrel, estradiol cypionate and estradiol valerate. Nystatin is an antifungal drug used for candidiasis treatment and no evidence related to preeclampsia was found. In this big group of drugs, desogestrel, ulipristal and lynestrenol are contraceptives. Moreover, a recent study shows that a reduction of 2-methoxyestradiol levels is related to clinical severity in preeclampsia [56,57] and its administration improves endothelial NO-synthase [30]. Similarly, in women with preeclampsia, the levels of estradiol, pregnenolone and estriol seem to be reduced [58]. Additionally, 2-methoxyestradiol is a metabolite of 17β-estradiol and therefore the presence of several forms of estradiol and estriol in our repurposed list seems to be a plausible finding. Regarding contraceptives, the situation is complex. Thadhani et al., found that the use of oral contraceptives before pregnancy could decrease the risk of developing gestational hypertension but not preeclampsia [59]. However, Farley et al. found no statistically strong association [60], while Magnussen et al., found a protective effect of oral contraceptives against preeclampsia [61]. The only strange finding involves mifepristone, a glucocorticoid hormone antagonist used to interrupt pregnancy. When pregnant rats under the administration of a nitric oxide synthase inhibitor (L-NAME) simulating preeclampsia symptoms are treated with mifepristone, an elevation of blood pressure was noticed post-partum [62].

In the other highly representative ATC category, alimentary tract and metabolism, we found the following drugs: ergocalciferol, loperamide, sibutramine, drostanolone propionate (all of these are found in the common 36 drugs), calcifediol, vitamin E, cholecalciferol, rolapitant and DL-alpha-tocopherol. From these drugs, ergocalciferol, calcifediol, DL-alpha-tocopherol and cholecalciferol (as well as the other drug found in the common 36, paricalcitol) are related to vitamin D or D3. Vitamin D is already known to be a drug for preeclampsia prevention and was used in our initial set from clinical trials. However, the finding of vitamin E is interesting. Vitamin E together with vitamin C or independently had been found to be protective for preeclampsia [63,64,65]. Paricalcitol is a synthetic analog of vitamin D, calcifediol is a metabolite of vitamin D3 and DL-alpha-tocopherol is a synthetic analog of vitamin E. On the other hand, drostanolone is an anabolic steroid similar to testosterone and this hormone is found to be elevated in preeclampsia [25,66,67,68,69]. Therefore, these drugs are not good candidates for preeclampsia treatment. These are connected with the AR receptor (which is highly important in our ranking of targets) and in a normal environment, the conversion of testosterone into estrogens is possible. However, in preeclampsia aromatase, mRNA and protein expression are decreased in the placenta, consequently reducing the metabolism of androstenedione and testosterone into estrogenic metabolites [67]. These findings indicate that in future works, it would be relevant not only to include off-targets related to hypertension but also to the estrogen/androgen metabolism in order to improve the predictions for this family of drugs. Moreover, sibutramine is used for obesity management and loperamide is used as antidiarrheal drug. However, both drugs have shown possible teratogenic effects [70,71].

We consider that the methodology proposed here for drug-repurposing is innovative. The identification of the proteins of interest is carried out using the known drugs under clinical trials for preeclampsia. Based on these proteins, a profile of predicted interactions was computed. Our strategy aims at the identification of new drugs for preeclampsia that have a similar profile of interactions with proteins to those already in use or in clinical trials for preeclampsia management. At the same time, our approach is designed to avoid negative effects of the repurposed drugs. To the best of our knowledge, this is one of the few attempts of accomplishing a completely computational approach for drug repurposing in preeclampsia.

## 3. Materials and Methods 

### 3.1. Candidate Drugs for Targtes and Off-Targtes Definition

Antihypertensive drugs following the mode of action through ACE inhibition and angiotensin receptor antagonist should not be used during pregnancy because of undesirable effects (i.e., teratogenic properties). Therefore, these targets should be avoided. The groups of targets associated with this type of drugs are labeled here as “Off-Targets”. A total of 41 antihypertensive drugs with those modes of actions and the corresponding targets were identified in DrugBank 5.1.8 [72]. These drugs are: azilsartan medoxomil, benazepril, benazeprilat, candesartan, candesartan cilexetil, captopril, cilazapril, cilazaprilat, delapril, enalapril, enalaprilat, eprosartan, fimasartan, forasartan, fosinopril, fosinoprilat, imidapril, irbesartan, lisinopril, losartan, moexipril, moexiprilat, olmesartan, omapatrilat, perindopril, perindoprilat, quinapril, quinaprilat, ramipril, ramiprilat, rescinnamine, saprisartan, saralasin, spirapril, tasosartan, telmisartan, temocapril, trandolapril, trandolaprilat, valsartan and zofenopril.

Additional targets were also extracted from ChEMBL version 27 [73] for these drugs as follows:

(1) All reported interactions with (IC50, Ki, EC50, GI50) were extracted from the ChEMBL database version 27.
(2) All extracted interactions were labeled as active if interaction values are lower than 10 μM and as inactive otherwise.
(3) If more than one report (active or inactive) was available for the same compound-target interaction, then the final criterion (active or inactive) was assigned considering 75% agreement to occur among reports. That is, if at least 75% of the reports did not agree on the same criterion, then the relevant compound was discarded.

From the ClinicalTrials database (https://clinicaltrials.gov/ct2/home), all records related to clinical trials for preeclampsia (prevention and/or treatment) were identified (a total of 283 records). A manual curation was done considering the following criteria: (1) Combined treatments were excluded, (2) treatments using proteins or inorganic salts were excluded, (3) all clinical trials evaluating drugs in post-partum or during labor conditions were excluded, (4) drugs in phase 1 or 2 (when information is available) were excluded and (5) when results data were present, drugs with negative results were excluded. A total of 19 drugs were finally selected: aspirin, labetalol, lidocaine, methyldopa, nicardipine, nifedipine, pravastatin, progesterone, sildenafil, diltiazem, coenzyme q10, arginine, vitamin D, azithromycin, docosahexaenoic acid, esomeprazole, urapidil, citruline and metformin. The same procedure described above for off-targets identification was applied to these 19 drugs, yielding 120 targets relevant for preeclampsia.

Additionally, a third database was created for drug repurposing using only the approved drugs identified in the DrugBank 5.1.8 database. This database comprises 2214 approved drugs.

### 3.2. Data Curation and Model Construction

From the 19 drugs selected for preeclampsia and the 41 drugs for off-targets analyses, we obtained 120 targets and 54 off-targets targets from CHEMBL and DrugBank. In total 155 unique proteins were found (155 instead 174 because there are several common proteins between targets and off-targets). For these 155 targets, we extracted all compounds from ChEMBL reporting active and inactive interactions with them, as described above. One dataset per protein including all compounds for which an interaction with it could be established was thus created. For all the compound-target interactions datasets, we performed the following curation steps sequentially: (1) removal of all of the 2214 approved drugs as well as the 13 and 41 drugs used for defining the targets and off-targets groups, (2) removal of all proteins with less than 100 active and 100 inactive compounds. These filters were applied to 55 total proteins and 147,456 compounds-proteins interactions. In this final dataset, there are 35 target, 10 off-targets and 10 targets common to both groups. The common targets were considered as “targets” and therefore 45 proteins were finally classified as “targets” and 10 proteins as “off-targets”.

All active/inactive compounds for each one of the 55 proteins can be found in Supplementary Materials (*chemblid_data.zip*, where each target is identified with its ChEMBL version 27 identification number).

Each of the 55 proteins was separately modeled as follows:

(1) Each compound was codified as a vector of 1024 features using the Morgan Circular Fingerprint of radius 4 using RDKit [74]

(2) For unbalanced datasets, the majority class (active or inactive) was reduced to obtain a balanced dataset. For this, a principal component analysis (on the 1024 descriptors) was performed. We kept as many principal components as needed to obtain an explained variance higher than 90% using the PCA function (*sklearn.decomposition*) from *scikit-learn* Python library. With the reduced number of variables, a clustering procedure using the K-means algorithm was carried out. The *KMeans* function from *sklearn.cluster* module available in the same Python library was used for clustering. K-mean was performed with a number of clusters in the range 3 – (nPCA+1), where nPCA is the number of principal components obtained in the previous step. For the selection of the optimal number of clusters, we used the maximum value of the Silhouette coefficient. For this computation, we used the function *silhouette_score* from *sklearn.metrics*. Finally, extraction of molecules matching the size of the minority class was done by using random selection from each cluster in proportion to the size of the cluster.

The balanced datasets for the 55 targets can be found in Supplementary Materials in the following files: *chemblid_data_training.zip* comprise all balanced data and *chemblid_data_notused.zip* that contains the samples that were removed after balancing the data. The python script *PartitionDataFile.py* was used to create these groups.

(3) Form each balanced dataset, 25% was randomly separated for external validation.

(4) The TPOT pipeline optimization was used for model constructions [75]. TPOT is an autoML method that includes data pre-processing, feature engineering (data scaling, feature union), feature selection and machine learning classifiers. We used *TPOTClassifier* (max_time_mins = 300, cv = 5) and accuracy as scoring metrics. Because each model is computed separately, the final pipeline was different for each model. The final model obtained for each target was used to compute its accuracy, precision, recall and f1-score on the external dataset.

All active/inactive compounds selected as external sample for each one of the 55 proteins can be found Supplementary Materials in the file *chemblid_data_training_external.zip*. Additionally, the python script *TPOTtestDataFile.py* was used to compute the external partition and to identify the optimal model from TPOT optimization. This pipeline optimization generates an optimal model for each target. The optimal models can be found in the *tpot_CHEMBLID.zip* file, which is the python script used for screening molecules against each target.

### 3.3. Virtual Screening Dataset and Procedures

The virtual screening dataset was constructed using: (1) The 19 drugs derived from ClinicalTrials under investigation for preeclampsia treatment and the 41 antihypertensive drugs (used to define off-targets), (2) all active compounds in the external dataset of all the Off-Target proteins and (3) all active compounds excluded in the data balancing for the creation of the training dataset of off-targets compounds (*chemblid_data_notused.zip*). Therefore, none of these molecules were involved in the model construction. This dataset for virtual screening comprises a total of 14,686 molecules and is provided as Supplementary Materials (*ScreeningData.txt*).

All of the 14,686 molecules were evaluated by all 55 models (45 for preeclampsia and 10 for off-targets which can be found in *tpot_CHEMBLID.zip*). However, we should keep in mind that probably not all targets have the same contribution to the virtual screening. For example, some targets and/or off-targets could be more promiscuous than others and consequently will have a high weight, leading to its inclusion or exclusion in the task of selecting the best drugs. Therefore, after having all models for the 55 proteins, a new question emerged: what is the best way to combine them in order to ensure the maximal recovery of the desirable drugs?

In order to explore the best group of target and off-targets yielding the best performance in virtual screening, we used genetic algorithms for feature selection (in this case, model selection). The genetic algorithm was run using the predictions of the 55 models across the 14,686 compounds. The population size for the genetic algorithm was set to 4000 individuals that evolve for 3000 generations with a mutation probability of 0.4. Individuals were codified as bit strings with a length of 55 bits, one bit per input model. A position was set to 0 or 1 if an individual excludes or includes, respectively, the corresponding target from the virtual screening aggregation. The objective function used of the genetic algorithm was based on a desirability function (Equation (1)) and BEDROC computation as described below.

First, the desirability of each compound (D_i_) was computed as:

(1) $$D_{i}=\sqrt{P_{i}^{P}P_{i}^{O}}$$

where:

$P_{i}^{P}=\frac{1}{N^{O}}\sum_{j}^{N^{P}}P_{i,j}^{P}$ and $P_{i}^{O}=\frac{1}{N^{O}}\sum_{j}^{N^{O}}\left(1-P_{i,j}^{O}\right)$, being:
N^P^ and N^O^ are the number of targets for preeclampsia and off-targets, respectively.
$P_{i}^{P}$ and $P_{i}^{O}$ are the average probabilities of compound “i” that are active against the targets of preeclampsia and off-targets, respectively.
$P_{i,j}^{P}$ and $P_{i,j}^{O}$ are the probabilities of the compound “i” that are active against target “j” in the preeclampsia and off-targets target lists, respectively.

The aforementioned computation took place across the models included in the individuals of the genetic individual population. Afterwards, the desirability scores were used as ranking criterion for the compounds and BEDROC with α = 160.9 (see below) was computed. Thus, BEDROC was maximized during the genetic algorithm evolution.

The virtual screening performance was evaluated by computing the Area Under the Accumulation Curve (AUAC), the Boltzmann-Enhanced Discrimination of ROC (BEDROC) and the Enrichment Factor (EF) [76,77]. In brief, let us consider the ranking of “N” compounds from which “n” are used in preeclampsia treatment. To each of these compounds corresponds a ranking r_i_ and a relative ranking x_i_ = r_i_/N in the whole ranked list. Given these definitions, the area under the accumulation curve AUAC was computed as:

(2) $$AUAC=1-\frac{1}{n}\sum_{i=1}^{n}x_{i}$$

The EF metric was defined as the number of desirable compounds found in a fraction 0<χ≤1 of the ordered list relative to what is expected from a random ranking for that same fraction:

(3) $$EF=\frac{\sum_{i=1}^{n}\delta_{i}}{\chi n},\;\mathrm{where}\;\delta_{i}=\left\{\begin{array}{c} 1\\ 0 \end{array}\;\begin{array}{c} r_{i}\leq \chi N\\ r_{i}>\chi N \end{array}\right..$$

The maximum value that EF can take is 1χ if χ≥nN and Nn if χ<nN and the minimum value is 0. Given that AUAC is based on the average position of the desirable compounds in the ranked list, it does not discriminate the early part of the ranked list from its last part. Likewise, EF does not take into consideration the position at which a desirable compound appears in the selected top ranked subset. Hence, these metrics were not appropriate to address the early recognition problem. For the evaluation of early recognition, the BEDROC metric was more appropriate and it was computed as:

(4) $$BEDROC=\frac{RIE-RIE_{\min}}{RIE_{\max}-RIE_{\min}}$$

with

$$RIE_{\min}=\frac{1-e^{\alpha R_{\alpha }}}{R_{\alpha }(1-e^{\alpha })},\;RIE_{\max}=\frac{1-e^{-\alpha R_{\alpha }}}{R_{\alpha }(1-e^{-\alpha })}\;\mathrm{and}\;RIE=\frac{\frac{1}{n}\sum_{i=1}^{n}e^{-\alpha x_{i}}}{\frac{1}{N}(\frac{1-e^{-\alpha }}{e^{\alpha /N}-1})}$$

In the above equations, Ra is the rate of desirables molecules in the dataset (n/N) and α is a parameter ensuring that active compounds ranked at the beginning of the ordered list get higher weights than those at the tail. The α parameter was computed from the equation:

$$\theta (1-e^{-\alpha })-1+e^{-\alpha z}=0$$

where z represents the fraction of the ranked list at which enrichment is important and θ is the expected contribution of the enrichment at this z% fraction to the overall enrichment. We set α = 160.9 that corresponds to giving a weight of 80% to the top 1% of the ranked dataset.

The python scripts *GA_VS_Model.py* and *GA_VS.py* were used for genetic algorithm optimization based on the probability prediction of each compound to each of the 55 targets (these probabilities can be found in *DesirabilityModels_F.txt*, Supplementary Materials). We executed the genetic algorithm optimization several times with different random initial populations. The results of the 4000 models can be found in Supplementary Materials (*DesirabilityModels_F_GA_Out.txt*).

### 3.4. Enrishment Analysis

When dealing with several genes, it is important to have a statistical strategy capable of establishing their relevance for specific biological processes or metabolic pathways. Enrichment analysis is the most used strategy to accomplish this task. We used ToppCluster [78] for this, which allowed us to explore enrichment analyses (in our case for metabolic pathways) across several groups simultaneously, thereby indicating common and uncommon terms between groups.

### 3.5. Targets Relevance and Global Desirability of Repurposed Drugs

The selection of the best multi-objective model for screening compounds using genetic algorithms produces several possible models. All models with BEDORC ≥ 0.15 (α = 160.9) were used to analyze the target relevance. However, not all models obtained with this BEDROC cutoff will necessarily include the same set of targets/off-targets. Therefore, in order to consider the possible heterogeneity of the obtained models, a clustering strategy was applied. A cluster analysis on the selected models in the final population of the genetic algorithm with best performance was done using the list of targets and off-targets as a fingerprint. The hierarchical clustering was done using the Euclidian distance with the identification of 4 and 7 clusters. Each model and cluster distribution can be found in Supplementary Materials (*ClustersInformations.txt*). The target/off target relevance was computed for both clustering strategies. The relevance of all targets (Targets and Off-targets) across each cluster was evaluated as: $S_{i}=\frac{1}{m}\sum_{j}^{m}T_{i,j}$ where m is the number of models and T_i,j_ is 1 if the target “i” was selected in the model “j” and 0 otherwise. In other words, the relevance of a particular targets is the normalized frequency of appearance of the target across all the models belonging to a particular cluster.

Using the 55 models, we computed the active/inactive probability of all approved drugs (see *DrugBank_Prob_Pred.txt* in Supplementary Materials). With those probabilities, we calculated the desirability (Equation (1)) for each of the multi-objective models in a particular cluster using the *DrugBankModelsClusters.py* script. This process yielded several desirability values for a particular drug (inside each cluster). The global desirability (GD_i_) of a drug in a particular cluster was computed by the average of all desirability in the cluster. This results in a consensus strategy that integrates the decisions of all multi-objective models rather than only the best model reported by the genetic algorithms in each cluster.

For each of the top selected drugs (50 per cluster), we identified their ATC codes (Level 1 and Level 2) using annotations in DrugBank 5.1.8, PubChem [79] and the WHO Collaborating Centre for Drug Statistics Methodology (https://www.whocc.no/).

## 4. Conclusions

The average accuracy of the models obtained for preeclampsia targets and off-target were 0.830 and 0.85 in the external dataset, respectively. We integrated all models using a desirability function and genetic algorithm to optimize the combination of models, leading to the bets initial enrichment in virtual screening. The performance metrics for the best performing model in virtual screening were: BEDROC = 0.258, EF1% = 31.55 and AUAC = 0.831.

The analysis of the final models points toward a relevance of AR, VDR, SLC6A2, NOS3 and CHRM4 as targets for preeclampsia. Similarly, the most relevant off-targets identified were: ABCG2, ERBB2, CES1 and REN. Among the top ranked repurposed drugs, estradiol, estriol, vitamins E and D, lynestrenol, mifrepristone, simvastatin, ambroxol and some antibiotics and antiparasitics seem to be safe and in some cases well supported in scientific literature as potential therapeutic alternatives for preeclampsia. On the other hand, the majority of the potential drugs identified herein remain completely unstudied focusing on their effects on pregnancy, preeclampsia or even placentation and we consider that they deserve further investigation.

## Data Availability

The data presented in this study are available in article and supplementary material.

## Supplementary Materials

Table S1: Performance metrics obtained in all 63 models. Table S2: Ranked drugs obtained from screening across all four clusters, DataAndModel.zip: This compressed file contains all data and python scripts described in the Material and Method section.

## Sample Availability

Samples of the compounds are not available from the authors.

## References

[1] Duhig K., Vandermolen B., Shennan A. (2018). Recent advances in the diagnosis and management of pre-eclampsia. F1000Research.

[2] Rolnik D.L., Wright D., Poon L.C., O’Gorman N., Syngelaki A., de Paco Matallana C., Akolekar R., Cicero S., Janga D., Singh M. (2017). Aspirin versus Placebo in Pregnancies at High Risk for Preterm Preeclampsia. N. Engl. J. Med..

[3] Vigil-De Gracia P., Ludmir J. (2015). The use of magnesium sulfate for women with severe preeclampsia or eclampsia diagnosed during the postpartum period. J. Matern. Neonatal Med..

[4] Mirzakhani H., Litonjua A.A., McElrath T.F., O’Connor G., Lee-Parritz A., Iverson R., Macones G., Strunk R.C., Bacharier L.B., Zeiger R. (2016). Early pregnancy vitamin D status and risk of preeclampsia. J. Clin. Investig..

[5] Serrano-Díaz N.C., Gamboa-Delgado E.M., Domínguez-Urrego C.L., Vesga-Varela A.L., Serrano-Gómez S.E., Quintero-Lesmes D.C. (2018). Vitamin D and risk of preeclampsia: A systematic review and meta-analysis. Biomedica.

[6] Stocks G. (2014). Preeclampsia. Eur. J. Anaesthesiol..

[7] Witcher P.M. (2018). Preeclampsia: Acute Complications and Management Priorities. AACN Adv. Crit. Care.

[8] Scaffidi J., Mol B., Keelan J. (2017). The pregnant women as a drug orphan: A global survey of registered clinical trials of pharmacological interventions in pregnancy. BJOG An Int. J. Obstet. Gynaecol..

[9] Goldstein J.A., Bastarache L.A., Denny J.C., Pulley J.M., Aronoff D.M. (2018). PregOMICS-Leveraging systems biology and bioinformatics for drug repurposing in maternal-child health. Am. J. Reprod. Immunol..

[10] Lotfi Shahreza M., Ghadiri N., Mousavi S.R., Varshosaz J., Green J.R. (2018). A review of network-based approaches to drug repositioning. Brief. Bioinform..

[11] Pushpakom S., Iorio F., Eyers P.A., Escott K.J., Hopper S., Wells A., Doig A., Guilliams T., Latimer J., McNamee C. (2018). Drug repurposing: Progress, challenges and recommendations. Nat. Rev. Drug Discov..

[12] Zhao S., Lv N., Li Y., Liu T., Sun Y., Chu X. (2020). Identification and characterization of methylation-mediated transcriptional dysregulation dictate methylation roles in preeclampsia. Hum. Genomics.

[13] Tejera E., Bernardes J., Rebelo I. (2012). Preeclampsia: A bioinformatics approach through protein-protein interaction networks analysis. BMC Syst. Biol..

[14] Tejera E., Cruz-Monteagudo M., Burgos G., Sánchez M.-E., Sánchez-Rodríguez A., Pérez-Castillo Y., Borges F., Cordeiro M.N.D.S., Paz-y-Miño C., Rebelo I. (2017). Consensus strategy in genes prioritization and combined bioinformatics analysis for preeclampsia pathogenesis. BMC Med. Genomics.

[15] Vaiman D., Calicchio R., Miralles F. (2013). Landscape of transcriptional deregulations in the preeclamptic placenta. PLoS ONE.

[16] Leavey K., Bainbridge S.A., Cox B.J. (2015). Large scale aggregate microarray analysis reveals three distinct molecular subclasses of human preeclampsia. PLoS ONE.

[17] Song Y., Liu J., Huang S., Zhang L. (2013). Analysis of differentially expressed genes in placental tissues of preeclampsia patients using microarray combined with the Connectivity Map database. Placenta.

[18] Kakigano A., Tomimatsu T., Mimura K., Kanayama T., Fujita S., Minato K., Kumasawa K., Taniguchi Y., Kanagawa T., Endo M. (2015). Drug Repositioning for Preeclampsia Therapeutics by In Vitro Screening: Phosphodiesterase-5 Inhibitor Vardenafil Restores Endothelial Dysfunction via Induction of Placental Growth Factor. Reprod. Sci..

[19] Sun M., Fan Y., Hou Y., Fan Y. (2018). Preeclampsia and maternal risk of breast cancer: A meta-analysis of cohort studies. J. Matern. Neonatal Med..

[20] Shibuya M. (2011). Involvement of Flt-1 (VEGF receptor-1) in cancer and preeclampsia. Proc. Jpn. Acad. Ser. B Phys. Biol. Sci..

[21] Todros T., Verdiglione P., Ogge G., Paladini D., Vergani P., Cardaropoli S. (2006). Low incidence of hypertensive disorders of pregnancy in women treated with spiramycin for toxoplasma infection. Br. J. Clin. Pharmacol..

[22] Alshareef S.A., Nasr A.M., Adam I. (2018). *Toxoplasma gondii* infection and pre-eclampsia among Sudanese women. Trans. R. Soc. Trop. Med. Hyg..

[23] Ambia A.M., Seasely A.R., Macias D.A., Nelson D.B., Wells C.E., McIntire D.D., Cunningham F.G. (2020). The impact of baseline proteinuria in pregnant women with pregestational diabetes mellitus. Am. J. Obstet. Gynecol. MFM.

[24] Ghaffari N., Gonzalez J.M., Rosenstein M.G. (2020). Does the 1-step method of gestational diabetes mellitus screening improve pregnancy outcomes?. Am. J. Obstet. Gynecol. MFM.

[25] Lan K.-C., Lai Y.-J., Cheng H.-H., Tsai N.-C., Su Y.-T., Tsai C.-C., Hsu T.-Y. (2020). Levels of sex steroid hormones and their receptors in women with preeclampsia. Reprod. Biol. Endocrinol..

[26] Naghshineh E., Sheikhaliyan S. (2016). Effect of vitamin D supplementation in the reduce risk of preeclampsia in nulliparous women. Adv. Biomed. Res..

[27] Tarca A.L., Romero R., Erez O., Gudicha D.W., Than N.G., Benshalom-Tirosh N., Pacora P., Hsu C.D., Chaiworapongsa T., Hassan S.S. (2020). Maternal whole blood mRNA signatures identify women at risk of early preeclampsia: A longitudinal study. J. Matern. Neonatal Med..

[28] Soobryan N., Murugesan S., Pandiyan A., Moodley J., Mackraj I. (2018). Angiogenic Dysregulation in Pregnancy-Related Hypertension—A Role for Metformin. Reprod. Sci..

[29] Saleh L., Verdonk K., Visser W., van den Meiracker A.H., Danser A.H.J. (2016). The emerging role of endothelin-1 in the pathogenesis of pre-eclampsia. Ther. Adv. Cardiovasc. Dis..

[30] Dong T., Sato S., Lyu J., Imachi H., Kobayashi T., Fukunaga K., Saheki T., Iwama H., Zhang G., Murao K. (2020). Treatment with 2-methoxyestradiol increases endothelial nitric oxide synthase activity via scavenger receptor class BI in human umbilical vein endothelial cells. Mol. Hum. Reprod..

[31] Cleophas M., Joosten L., Stamp L., Dalbeth N., Woodward O., Merriman T. (2017). ABCG2 polymorphisms in gout: Insights into disease susceptibility and treatment approaches. Pharmgenomics. Pers. Med..

[32] Moreno Santillan A.A., Briones Garduño J.C., Diaz de Leon Ponce M.A., Treviño-Becerra A., Iseki K. (2018). Uric Acid in Pregnancy: New Concepts. Contributions to Nephrology.

[33] Lye P., Bloise E., Nadeem L., Peng C., Gibb W., Ortiga-Carvalho T.M., Lye S.J., Matthews S.G. (2019). Breast Cancer Resistance Protein (BCRP/ABCG2) Inhibits Extra Villous Trophoblast Migration: The Impact of Bacterial and Viral Infection. Cells.

[34] Hou H., Geng M., Zhang R., Liu W., Wang J., Li J., Lin Y., Liu S., Wang Z., Guo H. (2019). Value of ABCG2 Q141K and Q126X genotyping in predicting risk of preeclampsia in Chinese Han women population. Pregnancy Hypertens..

[35] Valente F.M., De Andrade D.O., Cosenso-Martin L.N., Cesarino C.B., Guimarães S.M., Guimarães V.B., Lacchini R., Tanus-Santos J.E., Yugar-Toledo J.C., Vilela-Martin J.F. (2020). Plasma levels of matrix metalloproteinase-9 are elevated in individuals with hypertensive crisis. BMC Cardiovasc. Disord..

[36] Onal I.K., Altun B., Onal E.D., Kirkpantur A., Gul Oz S., Turgan C. (2009). Serum levels of MMP-9 and TIMP-1 in primary hypertension and effect of antihypertensive treatment. Eur. J. Intern. Med..

[37] Hamutoğlu R., Bulut H.E., Kaloğlu C., Önder O., Dağdeviren T., Aydemir M.N., Korkmaz E.M. (2020). The regulation of trophoblast invasion and decidual reaction by matrix metalloproteinase-2, metalloproteinase-7, and metalloproteinase-9 expressions in the rat endometrium. Reprod. Med. Biol..

[38] Chen J., Khalil R.A. (2017). Matrix Metalloproteinases in Normal Pregnancy and Preeclampsia. Progress in Molecular Biology and Translational Science.

[39] Yu W.Y., Hill S.T., Chan E.R., Pink J.J., Cooper K., Leachman S., Lund A.W., Kulkarni R., Bordeaux J.S. (2021). Computational drug repositioning identifies statins as a modifier of prognostic genetic expression signatures and metastatic behavior in melanoma. J. Invest. Dermatol..

[40] Gimenez N., Tripathi R., Giró A., Rosich L., López-Guerra M., López-Oreja I., Playa-Albinyana H., Arenas F., Mas J.M., Pérez-Galán P. (2020). Systems biology drug screening identifies statins as enhancers of current therapies in chronic lymphocytic leukemia. Sci. Rep..

[41] Rahimi R., Nikfar S., Abdollahi M. (2006). Meta-analysis finds use of inhaled corticosteroids during pregnancy safe: A systematic meta-analysis review. Hum. Exp. Toxicol..

[42] Vatti R.R., Teuber S.S. (2012). Asthma and pregnancy. Clin. Rev. Allergy Immunol..

[43] Murphy V.E., Namazy J.A., Powell H., Schatz M., Chambers C., Attia J., Gibson P.G. (2011). A meta-analysis of adverse perinatal outcomes in women with asthma. BJOG An Int. J. Obstet. Gynaecol..

[44] Martel M.J., Rey É., Beauchesne M.F., Perreault S., Forget A., Maghni K., Lefebvre G., Blais L. (2007). Use of short-acting β2-agonists during pregnancy and the risk of pregnancy-induced hypertension. J. Allergy Clin. Immunol..

[45] Katz O., Sheiner E. (2008). Asthma and pregnancy: A review of twto decades. Expert Rev. Respir. Med..

[46] Wang X., Mao M., Liu S., Xu S., Yang J. (2019). A comparative study of bolus norepinephrine, phenylephrine, and ephedrine for the treatment of maternal hypotension in parturients with preeclampsia during cesarean delivery under spinal anesthesia. Med. Sci. Monit..

[47] Ngene N.C., Moodley J. (2020). Pre-eclampsia with severe features: Management of antihypertensive therapy in the postpartum period. Pan Afr. Med. J..

[48] De Alwis N., Beard S., Mangwiro Y.T., Binder N.K., Kaitu’u-Lino T.J., Brownfoot F.C., Tong S., Hannan N.J. (2020). Pravastatin as the statin of choice for reducing pre-eclampsia-associated endothelial dysfunction. Pregnancy Hypertens..

[49] Rana S., Rajakumar A., Geahchan C., Salahuddin S., Cerdeira A.S., Burke S.D., George E.M., Granger J.P., Karumanchi S.A. (2014). Ouabain inhibits placental sFlt1 production by repressing HSP27-dependent HIF-1α pathway. FASEB J..

[50] El-Shourbagy M.A.A., El-Refaie T.A., Sayed K.K.A., Wahba K.A.H., El-Din A.S.S., Fathy M.M. (2011). Impact of seroconversion and antichlamydial treatment on the rate of pre-eclampsia among Egyptian primigravidae. Int. J. Gynecol. Obstet..

[51] Nascimento R.A., Possomato-Vieira J.S., Gonçalves-Rizzi V.H., Bonacio G.F., Rizzi E., Dias-Junior C.A. (2018). Hypertension, augmented activity of matrix metalloproteinases-2 and -9 and angiogenic imbalance in hypertensive pregnancy are attenuated by doxycycline. Eur. J. Pharmacol..

[52] Rustveld L.O., Kelsey S.F., Sharma R. (2008). Association Between Maternal Infections and Preeclampsia: A Systematic Review of Epidemiologic Studies. Matern. Child Health J..

[53] Tooke L., Riemer L., Matjila M., Harrison M. (2016). Antiretrovirals causing severe pre-eclampsia. Pregnancy Hypertens..

[54] Saums M.K., King C.C., Adams J.C., Sheth A.N., Badell M.L., Young M., Yee L.M., Chadwick E.G., Jamieson D.J., Haddad L.B. (2019). Combination Antiretroviral Therapy and Hypertensive Disorders of Pregnancy. Obstet. Gynecol..

[55] Hastie R., Ye L., Hannan N.J., Brownfoot F.C., Cannon P., Nguyen V., Tong S., Kaitu’u-Lino T.J. (2018). Disulfiram inhibits placental soluble FMS-like tyrosine kinase-1 and soluble endoglin secretion independent of the proteasome. Pregnancy Hypertens..

[56] Lee D.K., Nevo O. (2015). 2-Methoxyestradiol regulates VEGFR-2 and sFlt-1 expression in human placenta. Placenta.

[57] Tripathi V., Jaiswar S.P., Deo S., Shankhwar P. (2019). Association of 2-Methoxyestradiol (2ME) Plasma Levels with Clinical Severity Indices and Biomarkers of Preeclampsia. J. Obstet. Gynecol. India.

[58] Berkane N., Liere P., Lefevre G., Alfaidy N., Nahed R.A., Vincent J., Oudinet J.-P., Pianos A., Cambourg A., Rozenberg P. (2018). Abnormal steroidogenesis and aromatase activity in preeclampsia. Placenta.

[59] Thadhani R., Stampfer M.J., Chasan-Taber L., Willett W.C., Curhan G.C. (1999). A prospective study of pregravid oral contraceptive use and risk of hypertensive disorders of pregnancy. Contraception.

[60] Farley K.E., Huber L.R.B., Warren-Findlow J., Ersek J.L. (2014). The association between contraceptive use at the time of conception and hypertensive disorders during pregnancy: A Retrospective Cohort Study of Prams Participants. Matern. Child Health J..

[61] Magnussen E.B., Vatten L.J., Lund-Nilsen T.I., Salvesen K.Å., Smith G.D., Romundstad P.R. (2007). Prepregnancy cardiovascular risk factors as predictors of pre-eclampsia: Population based cohort study. Br. Med. J..

[62] Liao Q.P., Buhimschi I.A., Saade G., Chwalisz K., Garfield R.E. (1996). Regulation of vascular adaptation during pregnancy and post-partum: Effects of nitric oxide inhibition and steroid hormones. Hum. Reprod..

[63] Lorzadeh N., Kazemirad Y., Kazemirad N. (2020). Investigating the preventive effect of vitamins C and e on preeclampsia in nulliparous pregnant women. J. Perinat. Med..

[64] Meng W.Y., Huang W.T., Zhang J., Jiao M.Y., Jin L., Jin L. (2020). Relationship between serum vitamin E concentration in first trimester and the risk of developing hypertension disorders complicating pregnancy. Beijing Da Xue Xue Bao..

[65] Kasture V., Kale A., Randhir K., Sundrani D., Joshi S. (2019). Effect of maternal omega-3 fatty acids and vitamin E supplementation on placental apoptotic markers in rat model of early and late onset preeclampsia. Life Sci..

[66] Valdimarsdottir R., Wikström A.-K., Kallak T.K., Elenis E., Axelsson O., Preissl H., Ubhayasekera S.J.K.A., Bergquist J., Poromaa I.S. (2021). Pregnancy outcome in women with polycystic ovary syndrome in relation to second-trimester testosterone levels. Reprod. Biomed. Online.

[67] Keya S.L., Khanam N.N., Chowdhury A.A., Ripon R., Tasnim T., Sharmin A. (2019). Relationship between Free Testosterone and Preeclampsia. Mymensingh Med. J..

[68] Ibrahim Z.M., Kishk E.A., Elzamlout M.S., Elshahat A.M., Taha O.T. (2020). Fetal gender, serum human chorionic gonadotropin, and testosterone in women with preeclampsia. Hypertens. Pregnancy.

[69] Kumar S., Gordon G.H., Abbott D.H., Mishra J.S. (2018). Androgens in maternal vascular and placental function: Implications for preeclampsia pathogenesis. Reproduction.

[70] Källén B.A.J. (2014). Antiobesity drugs in early pregnancy and congenital malformations in the offspring. Obes. Res. Clin. Pract..

[71] Källén B., Nilsson E., Olausson P.O. (2008). Maternal use of loperamide in early pregnancy and delivery outcome. Acta Paediatr. Int. J. Paediatr..

[72] Wishart D.S., Feunang Y.D., Guo A.C., Lo E.J., Marcu A., Grant J.R., Sajed T., Johnson D., Li C., Sayeeda Z. (2018). DrugBank 5.0: A major update to the DrugBank database for 2018. Nucleic Acids Res..

[73] Bento A.P., Gaulton A., Hersey A., Bellis L.J., Chambers J., Davies M., Krüger F.A., Light Y., Mak L., McGlinchey S. (2014). The ChEMBL bioactivity database: An update. Nucleic Acids Res..

[74] (2018). RDKit, Open-Source Cheminformatics. http://www.rdkit.org.

[75] Le T.T., Fu W., Moore J.H. (2020). Scaling tree-based automated machine learning to biomedical big data with a feature set selector. Bioinformatics.

[76] Truchon J.-F., Bayly C.I. (2007). Evaluating virtual screening methods: Good and bad metrics for the “early recognition” problem. J. Chem. Inf. Model..

[77] Kirchmair J., Markt P., Distinto S., Wolber G., Langer T. (2008). Evaluation of the performance of 3D virtual screening protocols: RMSD comparisons, enrichment assessments, and decoy selection—What can we learn from earlier mistakes?. J. Comput. Aided. Mol. Des..

[78] Kaimal V., Bardes E.E., Tabar S.C., Jegga A.G., Aronow B.J. (2010). ToppCluster: A multiple gene list feature analyzer for comparative enrichment clustering and network-based dissection of biological systems. Nucleic Acids Res..

[79] Kim S., Chen J., Cheng T., Gindulyte A., He J., He S., Li Q., Shoemaker B.A., Thiessen P.A., Yu B. (2019). PubChem 2019 update: Improved access to chemical data. Nucleic Acids Res..

